# Natural Compounds with Antimicrobial Properties in Cosmetics

**DOI:** 10.3390/pathogens12020320

**Published:** 2023-02-15

**Authors:** Kamila Rybczyńska-Tkaczyk, Anna Grenda, Anna Jakubczyk, Kaja Kiersnowska, Marta Bik-Małodzińska

**Affiliations:** 1 Department of Environmental Microbiology, University of Life Sciences in Lublin, St. Leszczyńskiego 7, 20-069 Lublin, Poland; 2 Department of Pneumonology, Oncology and Allergology, Medical University in Lublin, ul. Jaczewskiego 8, 20-090 Lublin, Poland; 3 Department of Biochemistry and Food Chemistry, University of Life Sciences in Lublin, Skromna 8, 20-704 Lublin, Poland; 4 Institute of Soil Science, Engineering and Environmental Management, University of Life Sciences in Lublin, ul. Leszczyńskiego 7, 20-069 Lublin, Poland

**Keywords:** antimicrobial properties, cosmetics, polyphenols, plant extract, peptides, essential oils

## Abstract

Currently, the cosmetic industry is a very intensively growing part of the economy. Consumer demands are adapted to the current lifestyle, which is based on technological innovations and awareness of the impact of various factors on human health and fitness. There is growing interest in cosmetics based on environmentally friendly natural compounds exerting health-promoting effects. Chemicals with antimicrobial properties used as ingredients in cosmetics ensure their durability and safety. Polyphenolic compounds, peptides, essential oils, and plant extracts characterized by these properties are natural ingredients that can replace synthetic components of cosmetics. The advantage of these compounds is that they exhibit antioxidant, anti-inflammatory, and soothing properties, enhancing the product value in addition to their antimicrobial properties. This review article describes the antimicrobial properties of natural compounds that can protect cosmetics and can replace previously used preservative agents. Various studies indicate that the use of these compounds increases consumer interest in these products and has a positive impact on the environment.

## 1. Introduction

The globalization process and industrial development are increasingly being perceived as negative factors by consumers. Buyers make choices based on compliance with ecology, which involves the selection of natural products free of synthetic ingredients. Given the increasing awareness in society, producers compete with other companies in their contribution to the care of the environment. For a few years, new sustainable “eco-friendly” products have been evaluated for their potential use in many industry branches, e.g., food, clothes, and cosmetics. Particularly, the cosmetic area has rapidly improved its response to consumer needs [1]. Moreover, according to the World Health Organization report, microbial resistance to antibiotics has become a very serious health problem, especially due to the widespread use of these agents in food, health, and animal production. Due to the increased resistance of microorganisms to antibiotics, even a minor infection can be life-threatening. It is estimated that infections by antibiotic-resistant microorganisms cause 700,000 deaths per year worldwide. Therefore, scientists are still searching for new effective antimicrobial agents characterized by low toxicity, low production cost, and wide application in various areas [2].

The increase in the demand for natural compounds in products is accompanied by customer awareness of the negative effects of the use of synthetic preservative substances [1]. These ingredients play an essential role in preventing the growth of microorganisms and spoilage [3]. These include parabens such as alkyl esters of 4-hydroxybenzoic acid derived from synthetic esters of p-hydroxybenzoic acid, e.g., methyl paraben (MP), ethyl paraben (EP), n-propyl paraben (PP), benzyl paraben (BeP), isobutyl paraben (IBP), isopropyl paraben (IPP), n-butyl paraben (BP), and heptyl paraben (HP), and their respective sodium salts. They have been used as cosmetic, pharmaceutical, and food preservatives since the mid-1920s. Unfortunately, they can cause side effects such as hormone disruption via their mimicry effect on the endocrine system, diseases such as cancer, thyroid disorders, skin allergies, or even reproduction and neurological problems [4]. In addition, residues of these compounds present in domestic wastewater pollute freshwater, which disrupts aquatic [5] ecosystems. Therefore, new effective and low-toxicity preservative compounds that have no side effects on animal and human organisms or the environment are being sought.

The incorrect use of cosmetics by the consumer may produce serious health effects, e.g., eye infections, corneal ulcers, dermatitis, phlebitis, or folliculitis [6]. Hazardous microbiological contamination may occur at any stage of production or use of the product. Cosmetics contain water and organic and inorganic compounds, which favors the development of pathogenic microorganisms posing danger to consumers. Therefore, antimicrobial agents are used to increasing the durability and safety of cosmetics [7]. Noteworthy, the existing act on cosmetics imposes an obligation to test the microbiological purity of products to exclude the presence of the most common pathogens, i.e., *Staphylococcus aureus*, *Citrobacter freundii*, *Pseudomonas aeruginosa*, and *Candida albicans*. However, despite compliance with these recommendations, cosmetic products are often contaminated by microorganisms due to the poor awareness of the risk of contamination among both consumers and manufacturers [8].

To provide the safety of products, cosmetic science and industry have been searching for alternatives for synthetic compounds characterized by antimicrobial and antifungal action. Essential oils (EOs) obtained from plants with the use of extraction, distillation, or adsorption techniques are commonly used as preservatives in creams, shower gels, soaps, serums, etc., additionally serving as fragrance carriers. They generally contain several bioactive compounds with antimicrobial activity, e.g., terpenes, ethers, and alcohols [9]. As shown by literature data, tea tree oil (*Melaleuca alternifolia* var. *alternifolia*), rosemary oil (*Rosmarinus officinalis*), eucalyptus oil (*Eucalyptus globules* Labill.), thyme oil (*Thymus vulgaris* L.), lavender oil (*Lavandula angustifolia*), peppermint oil (*Mentha piperita* L.), and cinnamon oil *(Cinnamomum zeylanicum* J.Presl) are used most commonly in cosmetics. In addition to their antimicrobial activity, EOs exert warming, relaxing, and anti-inflammatory effects [1,10]. 

Enrichment of cosmetics with plant extracts is an alternative preservation process. These compounds are obtained in the process of methanol or water extraction, with the predominance of the former extractant [11]. It has been found that polyphenols, alkaloids, and saponins are constituents of various plant extracts, and phenols are considered to have the most potent antimicrobial and antifungal activity [7]. These plant extracts are derived from green tea leaves (*Camellia sinensis* (L.) Kuntze), marigold (*Calendula officinalis* L.), peppermint (*Mentha piperita* L.), narrow-leaved lavender (*Lavandula angustifolia*), Ceylon cinnamon (*Cinnamomum zeylanicum* J.Presl), thyme (*Thymus vulgaris* L.), cranberry fruit (*Vaccinium macrocarpon*), and cornflower (*Centaurea cyanus* L.) [12]. Furthermore, cosmetic science has improved rapidly, leading to the development of new alternatives to synthetic preservatives. As reported by some research data, silver nanoparticles [13] or methylglyoxal from manuka honey [3] are perceived as promising substances.

Various investigations are focused on the prevention of harmful microbiota growth and support of antibiotic treatment, given the increasing drug resistance of bacteria resulting from the use of antibiotics in medicine and food production. Increasing antibiotic resistance of preservative-tolerant bacterial strains isolated from cosmetic products can be observed as well [14]. As reported by Orús et al., cross-insensitivity to preservatives other than commonly used compounds and cross-insensitivity to antibiotics may develop. *Enterobacter gergoviae*, *Pseudomonas putida*, and *Burkholderia cepacia* were shown to be involved in recurrent contamination of cosmetic products containing preservatives. The researchers found reduced sensitivity of the isolates to formaldehyde but not to other common preservatives of cosmetics. They also showed increasing resistance of these strains to various antibiotics (β-lactams, quinolones, rifampicin, and tetracycline) compared to the wild-type strain [15]. Akgül et al. detected bacteria (e.g., *S. epidermidis*, *S. hominis*, *S. aureus*, *E. coli*, *K. pneumoniae*, and *P. aeruginosa*) in 20% of 500 cosmetic products used by consumers [16]. The authors found that one isolate of *S. aureus* was MRSA (methicillin-resistant) and 10 bacterial isolates exhibited resistance to carbapenem and extended spectrum beta-lactamase [16]. As shown in the study, cosmetic products may be a source of bacteria with acquired antibiotic resistance, which may be triggered indirectly by the use of human-made preservatives. Neza and Centini analyzed RAPEX (Safety Gate: the Rapid Alert System for dangerous non-food products) data from January 2008-2014 on microbiologically contaminated cosmetics and over-preserved cosmetic products. They observed that cosmetic product recalls due to the presence of pathogenic microorganisms involved approximately 11.8 percent of reports. *P. aeruginosa* was identified in the products most frequently, but mesophilic aerobic *Staphylococcus aureus*, *Candida albicans*, *Enterococcus* spp., *Enterobacter cloacae*, *Enterococcus faecium*, *Enterobacter gergoviae*, *Rhizobium radiobacter*, *Burkholderia cepacia*, *Serratia marcescens*, *Achromabacter xylosoxidans*, *Klebsiella oxytoca*, *Bacillus firmus*, *Pantoea agglomerans*, *Pseudomonas putida*, *Klebsiella pneumoniae*, and *Citrobacter freundii* were present as well. The researchers also indicated that the amounts exceeding the standard for such preservatives as methylisothiazolinone, benzalkonium chloride, and triclosan were responsible for the recall of approximately 2% of cosmetic products. Methyldibromo glutaronitrile, i.e., a substance prohibited in cosmetic products, was detected as well. These data indicate the need to protect cosmetics from harmful microbiota with the use of compounds of natural origin that will not affect the quality of cosmetic products, but will ensure microbiological safety at the production stage and effectively protect the product from secondary contamination.

Peptides produced by organisms in a free form or as part of proteins have also been applied as natural preservatives in cosmetic production. Given their various activities, they are also regarded to have a cleansing effect, support wound healing, and exhibit anti-inflammatory and antioxidant properties [2]. Currently, peptides are rarely used as ingredients in cosmetics due to the high cost of production and insufficient knowledge of their exact action. Therefore, new sources of bioactive peptides with potent antimicrobial properties and no side effects are still being sought.

This study is aimed to review the latest knowledge on natural substances with antibacterial and antifungal properties used in cosmetics.

## 2. Natural Compounds with Antimicrobial Properties as Cosmetic Ingredients

### 2.1. Plant Extracts

Extracts of different parts of plants (roots, stems, flowers, and fruits) are widely used for the inhibition of microbial growth [17]. They inhibit the growth of microorganisms responsible for primary and secondary microbial infections in cosmetics (Table 1). Therefore, in addition to their antimicrobial properties, they also have the potential to be used as preservatives [18,19]. At the production stage, semi-finished products of plant origin are mostly exposed to infection with microorganisms originating from water (most often *E. coli*) and living on plant material (e.g., *B. cereus*). During use, cosmetics are exposed to both bacteria and fungi, with *P. aeruginosa* or *S. aureus* bacteria and *C. albicans* or *A. niger* fungi as the most common infection agents at this stage [17,20,21]. 

Crude extracts of some spices and herbs, e.g., cinnamon (*Cinnamomum verum* J.Presl), garlic (*Allium sativum* L.), basil (*Ocimum basilicum* L.), ginger (*Zingiber officinale* Rosc.), sage (*Salvia officinalis* L.), roselle (*Hibiscus sabdariffa)*, rosemary (*Rosmarinus officinalis)*, clove (*Eugenia caryohyllata*), and thyme (*Thymus vulgaris* L.), have been reported to exhibit antimicrobial properties against a wide range of Gram-positive and Gram-negative bacteria [17,22,23,24,25]. The antibacterial activity of herb and spice extracts depends on the polarity of solvents used in the extraction process [26]. Liang et al. indicated that the use of low-polarity solvents (e.g., N-butane) or high-polarity solvents (e.g., ethanol) resulted in the extraction of lipophilic compounds from cinnamon (*Cinnamomum* sp.) or lipophilic and hydrophilic components, respectively. N-butane extracts of *C. cassia* and *C. loureiroi* exhibited much higher antibacterial activities than *C. wilsonii* and *C. burmannii* extracts. The minimum bactericidal concentrations (MBC) towards *Listeria monocytogenes*, *Staphylococcus aureus*, *Escherichia coli*, and *Salmonella anatum* ranged from 0.31 to 2.50 mg/mL in the case of n-butane *Cinnamomun* sp. extracts and from 20.00 to 160.00 mg/mL in the case of ethanol extracts [27]. 

Saffron is among the world’s most expensive spices with many health-promoting properties. The main medicinal and culinary applications have been found for *Crocus sativus* L. floral stigmas. *C. sativus* L. flowers have also been shown to exhibit many properties, including antibacterial activity. Belyagoubi et al. [28] determined the antimicrobial properties of extracts from saffron stigmata and flowers against seven bacterial and two *Candida* strains. As indicated by the results, the MIC values against *B. subtilis* and *M. luteus* were 781.25 and 6250 μg/mL for the flowers and 1406.25 and 22,500 μg/mL for the stigmata, respectively. In turn, the MBC of the flower extract was 100,000 μg/mL against *M. luteus* and ≥200,000 μg/mL against *B. cereus* and *B. subtilis*. As indicated by our previous results, *M. luteus* and *B. subtilis* were resistant to the stigma extract, with the best MBC value of 45,000 μg/mL. These studies of saffron extracts showed higher antimicrobial activity against Gram-positive than Gram-negative bacteria.

Atwaa et al. [29] investigated the possibility of using extracts from five medicinal plants (sumac, tamarind, rosemary, roselle, and lemon) against six microbial agents (*E. coli*, *P. *aeruginosa**, *B. subtilis*, *S. aureus*, *Penicillium* sp., and *A. niger*). These extracts can be used as substitutes for styptic antimicrobial compounds. The growth inhibition zone ranged from 14 to 45 mm. The tamarind and roselle extracts were characterized by growth inhibition zones in the range of 8–36 and 8–34 mm, respectively. Noteworthy, all these extracts were less active against fungi than against bacteria. In addition, all of the alcoholic extracts showed higher activity than the aqueous ones. The *B. subtilis* strain was the most sensitive to the plant extracts, while *P. aeruginosae* was the most resistant strain. The sumac extracts had the strongest antimicrobial properties with the MIC and MBC values ranging from 0.260 to 0.877 and from 0.310 to 1.316 mg/mL (antibacterial activity) and the MIC and MFC values ranging from 1.975 to 2.5 and from 2.5 to 4.444 mg/mL (antifungal activity), respectively. Therefore, extracts from these plants can be used as natural antimicrobial agents to eliminate pathogens.

*Hibiscus sabdariffa* L. has long been used in folk medicine to treat hypertension. The flowers of this plant are rich in bioactive compounds, e.g., polyphenols, flavonoids, tocopherols, or organic acids (malic acid, oxalic acid, and shikimic acid). Their composition and content varies depending on the extraction solvent used. It has been shown that hibiscus extracts can have antimicrobial activity against *E. coli* strains. Their antimicrobial properties depend on the solvent used for extraction. The aqueous extract of hibiscus showed moderate antimicrobial activity with an inhibition zone diameter of 18.33 mm. Noteworthy, the same extract exhibited the highest antimicrobial activity against *L. monocytogenes* strain ATCC 19115 with an average inhibition zone of 21 mm. In turn, the inhibition zone of hibiscus methanol extracts was 17.12 mm. The determination of the MIC and MBC/MFC values indicated that the hibiscus extracts in the liquid medium were highly active against all the tested bacteria and yeasts from the genera *Aspergillus*, *Fusarium*, and *Penicillium*, including *Aspergillus niger* DSM 63263, *Fusarium oxysporum*, *Penicillium expansum* DSM 994, *P. citrinum* DSM 1997, *P. simplicissimum* DSM 1097, *A. versicolor* DSM 1993, and *A. niger*. Four yeast strains, i.e., *Candida albicans* ATCC 2019, *C. parapsilosis* ATCC 22019, *C. kefyr* ATCC 6258, and *C. tropicalis* ATCC 06–085, were tested as well. The antibacterial activity of the hibiscus extracts was tested against nine bacterial strains frequently isolated from human infections and food poisoning: *Staphylococcus aureus* ATCC 25923, *S. epidermidis* CIP 106510, *Escherichia coli* ATCC 35218, *Listeria monocytogenes* ATCC 19115, *Pseudomonas aeruginosa* ATCC 27853, *Enterococcus faecalis* ATCC 29212, *Salmonella typhimurium* ATCC 1408, *Bacillus cereus* ATCC 11778, and *Vibrio parahaemolyticus* ATC 17802. In addition, these extracts were more effective against bacteria than against yeasts. In liquid cultures, the lowest MIC values were recorded for aqueous extract solutions (MIC: 9.375 mg/mL) and the lowest MFC was found for methanolic extracts (MFC: 18.75 to 37.5 mg/mL) [30]. Moreover, extracts from this plant were found to have soothing, softening, calming, and tonic properties [31]. 

Clove water extracts showed antibacterial activity against *S. aureus* and *E. coli* with MIC values of 2 and 2.5 mg/mL, respectively [23]. The problem of the rapid emergence of multidrug resistance (MDR) among bacteria, e.g., methicillin-resistant *S. aureus* (MRSA), *P. aeruginosa*, and *E. coli* causing potentially life-threatening infections, has been growing in recent years [32,33]. As suggested by Simões et al. [34], plant extracts or phytochemicals can be considered antimicrobials if their MIC determined during in vitro tests is in the range of 100–1000 μg/mL. Mandal et al. [33] reported the antibacterial activity of ethanolic extracts of *C. zeylanicum* J.Presl stem bark against clinical isolates of MRSA *S. aureus* (MIC = 64 µg/mL).

The antimicrobial properties of garlic extracts were analyzed by Gabriel et al. They reported the antibacterial activity of ultrasonicated garlic extract against *S. mutans*, *S. aureus sub. aureus*, *P. gingivalis*, and *E. coli* bacteria. As suggested by the researchers, these properties may result from the presence of phenolic compounds, organosulfur compounds, amino acids, carboxylic groups, and proteins in the analyzed extracts [35]. Water or toluene garlic extracts were described already in 1991 by Chowdhury or in 2005 by Rasmusen et al. as an effective agent in combating *Shigela flexnerii* or *P. aeruginosa* bacteria [36,37]. In their literature review, Bhatwalkar et al. indicated that the content and presence of active phytochemicals differed in garlic powder, fresh garlic juice, or extracts obtained with different extraction methods [38].

*S. mutans* bacteria present in the human oral cavity are causative agents of tooth decay. As indicated by Subramaniam et al., garlic extract-containing mouthwash preparations effectively eliminate this bacterium in the oral cavity [39]. Mouthwashes include chlorhexidine (CHX), which kills *Porphyromonas gingivalis*, i.e., a bacterium that can interfere with the stability of dental implants. Its presence may increase the risk of infection and soft tissue inflammation [40]. Interestingly, it has been shown that garlic extracts are effective in the fight against this bacterium and can be used as active ingredients in oral hygiene preparations [41,42]. Given the broad spectrum of their antibacterial activity, they can serve as natural preservatives of cosmetic/dental preparations. 

As shown by Yadav et al., aqueous garlic extracts have activity against *S. aureus* and *E. coli* [43]. The authors tested a concentration (*v*/*v*) of aqueous garlic extracts that provided the highest inhibition zone in the agar well diffusion method. They found that compared to ampicillin used against *E. coli* (inhibition zone 50 mm) and *S. aureus* (inhibition zone 70 mm), the largest zone of inhibition produced by the 100% concentration (*v*/*v*) was 34 and 37 mm, respectively [43]. The researchers concluded that the raw garlic extract exerted a strong antibacterial and antifungal effect and did not cause drug resistance, in contrast to the excessively used antibiotics or preservatives. Therefore, garlic extracts are worth considering for their use not only in the medical and pharmaceutical industry but also in the cosmetic industry as potential natural preservatives protecting against primary and secondary infections of cosmetic products. Fungal infections in the cosmetic industry are an important problem, as the multiplication of fungi in cosmetics is faster and more visible than bacterial growth [14]. Fungal contamination of cosmetics can be caused by, e.g., *Candida* spp., *Aspergillus* spp., and *Penicillium* spp. It can affect the quality of cosmetic products and reduce consumer safety [14,44,45]. Dadashi et al. found that 38.5% of powders and 30.0% of eyeliners were the most heavily fungus-contaminated products in shared cosmetic kits available in beauty salons [44]. Muhammad et al. reported *Penicillium * spp., *Aspergillus fumigatus*, and *Candida albicans* counts in the range of 102–104 CFU/mL in such cosmetics as mascaras, lip pencils, or eye pencils. Moreover, they concluded that water and other nutrients present in cosmetics make them susceptible to microbial growth. Garlic extracts showing antifungal activity may potentially replace strong antifungal substances used in the pharmaceutical and cosmetic industries. This was suggested by Pai and Platt, who showed that both aqueous extracts and concentrated garlic oil exerted similar or better inhibitory effects on *Aspergillus* species in comparison with pharmaceutical preparations and had similar minimal inhibitory concentrations [46]. 

The use of cosmetics may be associated with a risk of contracting dermatophyte infections. Liu et al. presented a case report on Majocchi’s granuloma (MG) caused by *Trichophyton rubrum* after facial hyaluronic acid injection [47]. As indicated by the authors, consumers are most concerned about such complications as infections and facial damage associated with injections of cosmetic products. The risk of infection with dermatophytes increases due to improper skin disinfection, incorrect injection techniques, reduced immunity, and the presence of pathogens [47]. Therefore, the safety of cosmetics in terms of the presence of bacteria and fungi seems to be particularly important also from the medical point of view, as infections associated with injections of cosmetic preparations may consequently create the need to use very strong antibacterial/antifungal drugs, as in the case of the MG patient. After a 2-month administration of 250 mg/d oral terbinafine, the patient still had painful papules, nodules, and abscesses on her face. Therefore, the itraconazole dose was adjusted to 400 mg/d for 8 weeks based on in vitro antifungal susceptibility testing results. The treatment turned out to be effective and alleviated the symptoms [47]. This case shows that fungal infections are difficult to treat and require very strong irritating agents. Aala et al. conducted studies on in vitro *T. rubrum* growth inhibition by allicin and garlic extracts [48]. This study showed that pure allicin (6.25 and 12.5 µg/mL) was more efficient in inhibition of the growth in hyphal cells than the garlic extracts (2 and 4 mg/mL) and both could be used as alternative agents in dermatophytosis treatment [48]. However, the doses used for the protection of cosmetic products against fungal infections may be different and perhaps the extract itself cannot bring satisfactory conservation effects and other preservatives must be added to the cosmetic. 

The antimicrobial/antifungal properties of basil extracts or oil have been extensively studied, and the results indicate their potential use as preservatives in food and cosmetics [49,50]. Crude or ethanol extracts are active against *S. aureus* and *E. coli*. Using a diffusion test, Khalil et al. found that ethanol extracts of basil leaves exhibited growth inhibitory activity against *E. coli* (21 mm inhibition zone) at 200 mg/mL and *S. aureus* (16 mm inhibition zone) at 200 mg/mL [51]. As reported by Taechowisan et al., *Ocimum basilicum* Linn. crude extract is an active agent against methicillin-resistant *Staphylococcus aureus* (MRSA) [52]. Using the diffusion method, the researchers determined the very high microbial activity of the crude extract at 34.5 mg/disc. Moreover, they noticed that the crude extract and the main isolated compound linalool showed higher activity against MRSA with MIC < 0.09 mg/mL and MBC ≤ 0.09 to 0.38 mg/mL than 1,8-cineole [52]. Kaya et al. conducted research on basil extracts obtained using various extractants: chloroform, acetone, and methanol (in two different water resuspension doses of 5 and 10 mL). Using a diffusion test, they examined the activity of individual extracts against *Enterococcus gallinarum*, *Enterococcus faecalis*, *Bacillus subtilis*, *Escherichia coli*, *Shigella* sp., *Streptococcus pyogenes*, *Staphylococcus aureus*, *Listeria monocytogenes*, and *Pseudomonas aeruginosa*. In addition, they checked the effect of basil extracts on *Saccharomyces cerevisiae Pakmaya* and *Candida* species. The methanol extracts had antimicrobial properties, while the chloroform and acetone extracts did not exhibit such activity. Furthermore, the methanol extracts produced inhibition zones against *Pseudomonas aeruginosa*, *Shigella* sp., *Listeria monocytogenes*, and *Staphylococcus aureus* strains and two different strains of *Escherichia coli*. Simultaneously, the 20 mL methanol/5 mL water extract seemed to have the best antimicrobial properties [53]. No antifungal properties of any of the extracts were noted in the research. Hossain et al. tested the antibacterial effect of essential oils and methanol extracts of sweet basil *Ocimum basilicum* L. (Lamiaceae) on the growth of food-borne pathogenic bacteria [54]. Using the disc method, they found that *O. basilicum* methanol extracts (300 μg/disc) had potential antibacterial activity against *Bacillus cereus*, *B. subtilis*, *B. megaterium*, *S. aureus*, *L. monocytogenes*, *E. coli*, *Shigella boydii*, *S. dysenteriae*, *Vibrio parahaemolyticus*, *V. mimicus*, and *Salmonella typhi* [54]. The inhibition zone for these bacteria ranged from 11.2 to 21.1 mm and the MIC values were in the range of 62.5–500 μg/mL [54]. Some of these bacteria can cause primary or secondary infections of cosmetics. The study conducted by Hossain et al. indicated the possibility of using natural compounds extracted from basil in the food and pharmaceutical industries and potentially in the cosmetic industry. Furthermore, the results showed a broad spectrum of activity of the compounds contained in basil; hence, basil extracts or oils are worth considering as potential natural preservatives.

**Table 1:** Plant extracts with antimicrobial properties.

Plant Extract	Strains	MBC	MIC	Reference
N-butane extract from cinnamon (*Cinnamomum* sp.)	*Listeria monocytogenes*, *Staphylococcus aureus*, *Escherichia coli*, and *Salmonella anatum*	Range from 0.31 to 2.50 mg/mL	nd	[27]
Ethanol extract from cinnamon (*Cinnamomum* sp.)	*Listeria monocytogenes*, *Staphylococcus aureus*, *Escherichia coli*, and *Salmonella anatum*	Range from 20.00 to 160.00 mg/mL	nd	[27]
Methanol:water extract from saffron (*Crocus sativus* L.) stigmata	*Bacillus subtilis * *Micrococcus luteus*	45,000 μg/mL 45,000 μg/mL	1406.25 μg/mL 22,500 μg/mL	[28]
Methanol:water extract from *Crocus sativus* L. flowers	*Bacillus subtilis * *Micrococcus luteus*	200,000 μg/mL 100,000 μg/mL	781.25 μg/mL 6250 μg/mL	[28]
Methanolic sumac extracts	*Escherichia coli*, *Pseudomonas aeruginosa*, *Bacillus subtilis*, *Staphylococcus aureus*, *Penicillium* sp., and *Aspergillus niger*.	Range from 0.310 to 1.316 mg/mL	Range from 0.260 to 0.877	[29]
Clove water extract	*Staphylococcus aureus* *Escherichia coli*	nd	2 mg/mL 2.5 mg/mL	[23]
Ethanolic extracts of *Cinnamomum zeylanicum* J.Presl	Methicillin-resistant *Staphylococcus aureus* (MRSA)	nd	64 µg/mL	[33]
Aqueous extract from basil (*Ocimum basilicum*) leaves	Methicillin-resistant *Staphylococcus aureus* (MRSA)	≤0.09 mg/mL	<0.09 mg/mL	[52]
*Ocimum basilicum* methanol extracts	*Bacillus cereus*, *B. subtilis*, *B. megaterium*, *Staphylococcus aureus*, *Listeria monocytogenes*, *Escherichia coli*, *Shigella boydii*, *Shigella dysenteriae*, *Vibrio parahaemolyticus*, *Vibrio mimicus*, and *Salmonella typhi*	nd	62.5–500 μg/mL	[54]

MBC—minimum bactericidal concentrations; MIC—minimum inhibitory concentration; nd—not determined.

Pharmaceutical properties of basil related to the content of polyphenols, phenolic acids, and flavonoids were also described by Romano et al. [55]. The researchers considered natural compounds used as preservatives in cosmetics. The method of extraction of these compounds or their mixtures in extracts or oils appeared to be important. As pointed out by Kaya et al. [53], not every extraction method will provide the desired composition and properties of extracts. Romano et al. [55] applied basil leaf extraction using carbon dioxide and ethanol as co-solvents. It turned out that the use of 10% ethanol as a co-solvent was the most efficient method, with a yield similar to that of the control (100% ethanol) and with significantly higher contents of caffeic acid (1.69–1.92 mg/g), linalool (35–27%), and bergamotene (11–14%) than those in the control [55]. As indicated by the authors, the supercritical CO_2_ extraction method not only provides extracts but is also environmentally friendly and contributes to reduced consumption of ethanol. 

Ueda et al. showed the potential of the use of basil and sage extracts as natural preservatives. They obtained plant extracts using ultrasound and an ethanol/water solution (80:20, *v*/*v*). The extracts were analyzed in terms of their composition and then added to yoghurt for further screening of its nutritional and physicochemical properties and the microbiological load for a shelf life of 14 days. The plant extracts were not hepatotoxic, did not induce changes in the physicochemical and nutritional properties of the yoghurt, and did not interfere with the growth of lactic acid bacteria [49]. The study conducted by Ueda et al. was focused on food products, but the strategy can be transferred to the field of cosmetic research. It is also necessary to check whether plant extracts used as natural preservatives are non-toxic, may cause allergic reactions, or change the natural human microbiota after the application of creams or other cosmetic products.

### 2.2. Phenolic Compounds

Phenolic compounds, i.e., among the most diverse secondary metabolites in plants, are a double-edged sword. On the one hand, they have mutagenic and genotoxic properties; on the other hand, they may prevent certain lifestyle diseases [56,57]. Antimicrobial properties are among the multiple activities of phenolic compounds naturally occurring in plants. They can be used in the food, pharmaceutical, or cosmetic industries. The group of phenolic compounds includes phenolic acids, flavones, lignans, and tannins [58]. 

Phenolic compounds exhibit a broad spectrum of activity. For example, protocatechuic acid (PCA, 3,4-dihydroxy benzoic acid) exerts antioxidant, anticancer, antiulcer, antidiabetic, antiaging, antifibrotic, antiviral, anti-inflammatory, analgesic, antiatherosclerosis, cardiac protective, hepatoprotective, neurological, nephroprotective, and antibacterial effects [59]. PCA is active against Gram-positive and Gram-negative bacteria as well as fungi. It is also involved in synergistic interactions with some antibiotics against resistant pathogens [59,60,61]. Jalali et al. indicated catechuic acid as an antiseptic agent with broad-spectrum activity against bacteria associated with surgical skin inflammation, including drug-resistant microorganisms, and with dose-dependent activity against *Cutibacterium acnes* [62]. As reported by Syafni et al., 3,4-dihydroxybenzoic acid (protocatechuic acid) isolated from leaves, stems, and roots of *Trichomanes chinense* L. fern (*Hymenophyllaceae*) has dose-dependent growth inhibitory activity against *E. coli*, *S. aureus*, and *S. typhimurium* [63]. The ability of phenolic compounds to inhibit *E. coli*, *K. pneumoniae*, *A. flavus*, *A. parasiticus*, and *B. cereus* growth was studied by Aziz and co-workers [64]. They found that caffeic and protocatechuic acids used at a concentration of 0.3 mg/mL inhibited *Escherichia coli* and *Klebsiella pneumoniae* growth. p-Hydroxy benzoic, vanillic, caffeic, protocatechuic, and p-coumaric acids as well as oleuropein and quercetin analyzed at a concentration of 0.5 mg/mL completely inhibited *Bacillus cereus* growth. Oleuropein and p-hydroxy benzoic, vanillic, and p-coumaric acids (0.4 mg/mL) inhibited the growth of *E. coli*, *K. pneumonia*, and *B. cereus*. Vanillic and caffeic acids (0.2 mg/mL) completely inhibited the growth and aflatoxin production by *A. flavus* and *A. parasiticus* [64]. As indicated in the study, phenolic compounds can inhibit the growth of microorganisms that cause secondary infections in cosmetics. Kim et al. analyzed an important branch of production of so-called customized cosmetics, which are designed and manufactured to meet individual customer needs [14]. Heat treatment and the point of transfer of the cosmetic to a new container were identified in the study as determinants of the presence of microorganisms or their increased content. Sequencing analyses of isolates contained in personalized cosmetic products revealed the presence of bacteria and moulds., i.e., human pathogens *S. epidermidis*, *B. cereus*, *B. circulans*, and *A. versicolor* [14]. Phenolic compounds were shown to be valuable additives to personalized cosmetics serving as effective and natural agents to prevent the growth of harmful microbiota (Table 2). 

With their antimicrobial activity, naturally occurring plant phenolic compounds are very attractive to the cosmetic industry. Due to the potential harmfulness of synthetic preservatives contained in cosmetics and their negative impact on human health, natural compounds that can eliminate harmful bacteria from cosmetic products are increasingly being studied. Phenolic compounds seem to be good candidates for antibacterial agents used in cosmetics. However, due to the rather broad spectrum of their activity, it is necessary to conduct thorough research on the action and allowable doses of individual compounds that can protect cosmetic products against the growth of destructive microbiota but do not change their properties. 

The antimicrobial and antifungal properties of phenolic acids are widely studied. A study conducted by Qian et al. focused on the use of vanillic acid as an antimicrobial agent against carbapenem-resistant *Enterobacter hormaechei* (CREH) [65]. Their results showed that this acid was effective against CREH with a minimum inhibitory concentration of 0.8 mg/mL. The acid caused the cell wall to rupture and thus destroyed the cell. In addition, vanillic acid effectively inhibited biofilm formation by CREH. As indicated by the researchers, this phenolic acid can potentially be used as a preservative and disinfectant. Gallic acid or caffeic acid are other phenolic acids with antimicrobial activities. Lima et al. determined the antibacterial, antifungal, and antibiotic modulatory activities of gallic acid, caffeic acid, and pyrogallol. It was found that pyrogallol reduced the required antibiotic concentration necessary to kill the entire *Candida albicans* population and, similar to gallic acid, was able to enhance antibiotic activity against *Staphylococcus aureus* [66]. In addition to phenolic acids, other phenolic compounds with antimicrobial effects may serve this purpose. Jaisinghani et al. indicated antimicrobial properties of quercetin against *S. aureus* and *P. aeruginosa* at a concentration of 20 µg/mL and *P. vulgaris* and *E. coli* at concentrations of 300 and 400 µg/mL, respectively. Moreover, *Shigella flexneri* and *Lactobacillus casei var. shirota* were indifferent even at a concentration of 500 µg/mL. [67]. As reported by Yang et al., quercetin has a broad spectrum of antimicrobial activity targeted at the destruction of bacterial cell walls and inhibition of nucleic acid synthesis [71]. The authors listed several bacteria identified in cosmetic products against which quercetin exhibited antibacterial properties. They described the activity of quercetin in clinical applications. The compound was shown to exert an inhibitory effect against drug-resistant *E. coli* or carbapenem-resistant *Pseudomonas aeruginosa*, i.e., bacteria with acquired antibiotic resistance, which are increasingly being identified in cosmetics, as indicated by Orús et al. [15]. Great interest in the medical field is aroused by antibacterial agents other than antibiotics due to the increasing emergence of drug-resistant bacteria. In this field, polyphenolic compounds and their antimicrobial properties are being assessed for use in cosmetics, given the emergence of resistant strains caused by the overuse of strong artificial preservatives. Research on the use of non-antibiotic compounds to prevent biofilm formation by *S. aureus* causing infections of implanted medical devices was conducted by Di Ming et al. [68]. The authors used kaempferol as an agent against *S. aureus* biofilm formation. They showed that kaempferol applied at a concentration of 64 μg/mL inhibited biofilm formation by 80%. The minimum inhibitory concentration and growth curve assays showed that kaempferol did not exhibit antimicrobial activity against *S. aureus*. They demonstrated that kaempferol was able to inhibit the primary attachment phase in biofilm formation and decreased *S. aureus* sortaseA (SrtA) activity and adhesion-related gene expression [68]. Such studies are highly important for cosmetics production, given the possible contamination and formation of bacterial biofilms on the surfaces of tools used for the preparation of cosmetics, especially in the case of the increasingly popular handmade or personalized natural products, where no strong synthetic bactericides are used in the production process. This may lead to the emergence of cross-resistance of bacteria to drugs and common preservatives used in industry. Nevertheless, more research is needed to investigate the use of natural plant phenolic compounds as antimicrobial substances in cosmetic products. As shown by Kumar et al., polyphenolic compounds kaempferol and combretastatin extracted from plant sources have antimicrobial activity [72]. However, this activity is limited due to its large size and insolubility in water. The researchers indicated that the nanoformulation of these compounds potentially inhibited bacterial growth. Their possible mechanism of bacterial growth inhibition may be related to the activation of the ATP-dependent efflux pump system in bacteria or the blocking of porin channels by nano-sized metabolism-disrupting compounds, thereby inhibiting bacterial growth [72]. Karpiński et al. reported that apigenin and luteolin MIC against *E. coli* and *P. aeruginosa* was 500 μg/mL. *S. aureus* strains showed weak sensitivity to apigenin (MIC = 500–1000 μg/mL) and were resistant to its derivatives vitexin and isovitexin (>1000 μg/mL). Additionally, these compounds weakly inhibited the growth of *E. faecalis* (1000 μg/mL). In contrast, luteolin and its C-glucosides (orientin, isoorientin) reached the same MIC values: moderate against *S. aureus* (500 μg/mL) and weak against *E. faecalis* (1000 μg/mL). The researchers indicated that apigenin, luteolin, and their C-glucosides were generally more potent against Gram-negative than Gram-positive bacteria [69]. It has been demonstrated that lignans, occurring naturally in plants, also have antimicrobial properties and are regarded as natural ingredients that can prevent the growth of harmful microbiota in consumer products. Favela-Hernández et al. reported strong antimicrobial properties of lignans isolated from *Larrea tridentata*. Their study demonstrated that 3′-demethoxy-6-O-demethylisoguaiacin lignan was active against sensitive and methicillin-resistant *S. aureus* (MIC 25 µg/mL), *E. faecalis* (MIC 12.5 µg/mL), *E. coli* (MIC 50 µg/mL), and *E. cloacae* (MIC 12.5 µg/mL). The researchers indicated that the lignan also showed efficacy with MIC 12.5 µg/mL against methicillin-resistant *S. aureus* isolated from clinical samples. In addition, dihydroguaiaretic acid and 4-epi-larreatricin lignans were active against *S. aureus* MR (MIC 50 µg/mL) and *E. cloacae* (MIC 12.5 µg/ mL), respectively [70]. Similarly, Rahmana et al. reported antimicrobial properties of lignans isolated from *Zanthoxylum budrunga* Wall. The two lignans exhibited antibacterial properties against *Staphylococcus aureus*, *Escherichia coli*, and *Proteus vulgaris* and antifungal activity against *Aspergillus niger* and *Candida albicans* with MIC values in the range of 0.06–0.239 µmol [73]. 

Phenolic compounds are a very broad group of naturally occurring plant compounds. The phenolic compounds presented here have been tested for their antibacterial or antifungal properties, which are very desirable nowadays in the face of the increasing occurrence of opportunistic microorganisms and the demand for natural consumer products, including cosmetics, with the potential to eliminate strong artificial preservative compounds. 

### 2.3. Essential Oils (EOs)

#### 2.3.1. General Information

The use of essential oils in various fields, especially in medicine, perfume, and cosmetic products, has been known practically since antiquity. Due to the growing popularity of natural products among consumers, the demand for the production of essential oils has increased. Essential oils are a very important component of plant material. They are often extracted from food production waste with a variety of methods. The content, quality, and properties of essential oils are influenced not only by the species, part of the plant, growth stage, and extraction method but also by the method of plant cultivation, processing, and storage [74].

Essential oils constitute a highly volatile lipophilic aromatic fraction of the phytocomplex containing tens to hundreds of different compounds, mainly terpenes and phenolic compounds. Essential oils are named after the plant from which they are isolated. The fragrance is more concentrated in the oil than in plant organs from which it was isolated [75].

EOs have been shown to exert many beneficial effects; hence, their components are widely used in the cosmetic industry. In addition to imparting a distinctive aroma to products, they may exhibit analgesic, antibacterial, diuretic, antioxidant, or anti-inflammatory properties. EOs can be used as preservatives in cosmetics due to their antimicrobial properties [76]. They are used as adjuvants to other preservatives or as the sole preservative in cosmetic products. Various studies have indicated that EOs exhibit higher antimicrobial activity when combined with other preservatives, chelating agents, and stabilizers. To be used as preservatives in cosmetics, EOs must be safe for the consumer, have no toxicity, and exhibit high activity in low concentrations. Their effect should be targeted toward a broad spectrum of microorganisms, and they should not have a dominant odor, taste, or color. EOs can be used as preservatives in almost all types of cosmetics.

#### 2.3.2. EOs as Natural Preservatives

Essential oils are used as ingredients in cosmetics, giving them biological properties, taste, and aroma. They can also serve as natural antimicrobial agents (Table 3). The most commonly used oils in cosmetics are derived from mint [77], chamomile [78], lavender [79] herbs and peach [80] or lemon [7] fruits.

Essential oils can also be extracted from other less common plants, e.g., *Ferulago stellata* Boiss. fruits. Various *Ferulago* species have long been used in traditional medicine for their sedative, tonic, and digestion-regulating properties. Their essential oil is used as a preservative in the production of meat, oil, or dairy products, additionally giving these products a characteristic pleasant taste [81].

Rahimpour et al. [81] studied the antimicrobial activity of *Ferulago* essential oil. It was evaluated by measuring growth inhibitory zones against four Gram-negative bacteria (*Escherichia coli*, *Enterococcus faecalis*, *Salmonella typhi*, *Pseudomonas aeruginosa*), three Gram-positive bacteria (*Staphylococcus epidermidis*, *Staphylococcus aureus*, *Listeria. monocytogenes*), and one fungus (*Candida albicans*) with the disc diffusion method. In general, the tested essential oil showed high antimicrobial activity against both Gram-negative and Gram-positive bacteria. In terms of MIC and MBC, the highest activity was achieved against *E. coli* (MIC = 0.78 μg/mL, MBC = 1.56 μg/mL), *P. aeruginosa* (MIC = 0.78 μg/mL, MBC = 1.56 μg/mL), *L. monocytogenes* (MIC = 0.78 μg/mL, MBC = 1.56 μg/mL), and *Candida albicans* (MIC = 0.78 μg/mL, MBC = 1.56 μg/mL). The study showed that the essential oil was less active against *E. faecalis* (MIC = 3.12 μg/mL, MBC = 6.25 μg/mL), as indicated by the smallest inhibition zone (19 ± 0.50 mm) obtained against this bacterium.

*Lavandula angustifolia* Mill. (syn. *Lavandula vera* DC, syn. *Lavandula officinalis* Chaix ex Vill., syn. *Lavandula spica* L.) is a very common intensely aromatic plant growing in Europe, North Africa, the United States, and Australia [87]. The composition of its oils depends on the method and conditions of cultivation. Ciocarlan et al. [88] determined the composition of seven samples of essential oil extracted from lavender by different manufacturers. The content of the ingredient varied between the samples. Monoterpenes (84.08–92.55%), including sesquiterpenes (3.30–13.45%), and some aliphatic compounds (1.42–3.90%) were the main fractions. As shown by the results, the lavender essential oil was characterized by high antibacterial activity against *Bacillus subtilis*, *Pseudomonas fluorescens*, *Xanthomonas campestris*, *Erwinia carotovora* with the minimal bactericidal concentration of 300 µg/mL, and *Erwinia amylovora* with the MBC value of 150 µg/mL; the minimal fungicidal concentration against *Candida utilis* was 150 µg/mL.

Essential oils may have various applications in cosmetic production, as they improve dermo-cosmetic properties, have an effect on skin firmness and rejuvenation, and above all exhibit preservative properties contributing to the final image of cosmetics as eco products. Tropical *Artemisia afra* and *Pteronia incana* plants and two Mediterranean aromatic plants grown in South Africa, i.e., *Lavandula officinalis* and *Rosmarinus officinalis*, were the sources of essential oils used in cosmetics as potential preservatives of creams with activity against *Escherichia coli*, *Staphylococcus aureus*, *Pseudomonas aeruginosa*, *Candida albicans*, *Aspergillus niger*, and two environmental isolates identified as *Ps. aeruginosa* and *Ralstonia pickettii*. It was shown that, except for the *Pseudomonas aeruginosa* strains, the oils had potent antimicrobial activity against all commonly tested organisms (including bacteria and fungi) and environmental isolates. All of the analyzed microorganisms were generally more sensitive to the oils during the challenge test in water cream compared to the agar antibacterial test. Thus, oils can be recommended as candidates for natural cosmetic preservatives. The *Artemisia afra* oil was the most effective in reducing a load of artificial impurities in an aqueous cream formulation within 2–7 days. Since they were used in relatively high concentrations, the oils were also reported to act as natural fragrances in the water cream and provide protection against microbial contamination [73].

Due to their aroma, taste, and high content of essential oils, roses have long been used for the production of food and cosmetics. The content, composition, and properties of their essential oils depend on the growing region. The yield and chemical composition of essential oils obtained from *Rosa* × *damascena* were found to depend significantly on the place of collection. In particular, the oil yield ranged from ~0.08 to ~0.132%, and citronellol (36.70–9.18%), geraniol (12.82–0.47%), nonadecane (22.73–10.36%), heneicosan (31.7–11.43%), and 1- nonadecane (6.03–3.93%) were detected as the major compounds present in different concentrations in all harvested plants. The inhibitory zone was 17.33 ± 0.58 mm against *Aspergillus brasiliensis*, 15.67 ± 0.58 mm against *Staphylococcus aureus*, and 12.33 ± 0.58 mm against *Streptococcus pyogenes*. The *R. damascena* essential oils were also effective against Gram-negative *Pseudomonas aeruginosa* with MIC values of 62.50 μg/mL, regardless of the harvest area. The extracted essential oil also exhibited antifungal potency against *Candida albicans* yeasts (MIC and MBC ~62.50 μg/mL) [83].

Essential oils may also show synergism with other compounds with antimicrobial properties. They are also produced by plants of the *Asteraceae* family, e.g., *Baccharis coridifolia*. As shown by some research, the essential oil extracted from this species has pharmacological properties and can be a promising source of new antimicrobial agents. Germacrene D (23.7%), bicyclermacren (17.1%), and (E)-caryophyllene (8.4%) were the main active substances identified in *Baccharis coridifolia* EO. Its minimum inhibitory concentration was 512 μg/mL against *Pseudomonas aeruginosa* strains and 128 μg mL against *Staphylococcus aureus*, and its antibacterial activity was regarded as clinically relevant. Moreover, sub-inhibitory doses of the oil combined with conventional antibiotics showed synergism and enhanced the antibacterial effect. This indicates that *Baccharis coridifolia* essential oil has antibacterial and antibiotic-modulating activity, which makes this species a useful source of molecules for combating bacterial resistance [89].

The antimicrobial properties of essential oils are also associated with plant organs. *Salvia hydrangea* from the *Lamiaceae* family has been widely used in traditional Iranian medicine. A study comparing the composition and antimicrobial properties of essential oils extracted from leaves or flowers of this plant indicated that their composition varied between the plant parts: (+)-spathulenol (16.07%), 1,8-kineol (13.96%), trans-caryophyllene (9, 58%), β-pinene (8.91%), and β-eudesmol (5.33%) were the most abundant bioactive substances contained in the essential oil extracted from the leaves, whereas the floral essential oil contained the greatest amounts of caryophylene oxide (35.47%), 1,8-kineol (9.54%), trans -caryophylene (6.36%), β-eudesmol (4.11%), caryophylenol II (3.46%), and camphor (3.33%). Both oils exhibited significant inhibitory and lethal activity against Gram-negative bacteria *Pseudomonas aeruginosa* (MIC ~16 µg/mL), *Shigella dysenteriae*, and *Klebsiella pneumoniae* (MIC ~62 µg/mL). This suggests that essential oils from *S. hydrangea* leaves and flowers may have potential applications as bactericides against some bacteria and can be used as cosmetic ingredients [90].

As indicated by numerous research results, essential oils not only give a characteristic desirable scent to cosmetics but can also be used as natural preservatives in various types of cosmetic products. Thus, cosmetics become more environmentally friendly and are more often chosen by consumers who prefer eco products.

### 2.4. Antimicrobial Peptides (AMPs)

#### 2.4.1. AMP Activity

Until recently, bioactive peptides were believed to consist of 2 to 3 amino acid residues [91], but it is currently known that they may contain a greater number of residues [92]. Peptides are produced by animals, plants, fungi, and microorganisms and occur in a free state. Proteins are the main precursors of peptides, which are most often released through enzymatic hydrolysis and serve many functions. They have been applied in many fields: food technology, pharmacy, medicine, and the cosmetic industry and play an important role in the functioning and regulation of biological processes.

Antimicrobial peptides (AMPs) are produced by all organisms. including microbes. as a first line of natural defense against pathogens [93]. They exhibit a wide spectrum of activity, should be low in toxicity and have a unique mechanism of bacterial membrane destruction [94]. Therefore, new AMPs are sought to be widely used and solve the problem of antibiotic resistance of pathogens. Numerous scientific studies provide data on new AMPs, but their application raises some doubts. The complicated procedure for the purification and isolation of these compounds is associated with high research and development costs, low stability [95], and bacterial resistance to AMPs [96], which limits their use [94].

AMPs show broad activity against bacterial, viral, and fungal pathogens or cancer cells. Moreover, they may have indirect activity by modulation of the host immune defense system to neutralize infection. These compounds exhibit similar activity to that of cytokines and growth factors, which are important elements in the maintenance of immune homeostasis. Additionally, they can prevent the development of infections by neutralizing Pathogen-Associated Molecular Patterns (PAMPs), especially lipopolysaccharide (LPS) and lipoteichoic acid (LTA) [93].

Noteworthy, AMPs have broad activity against both classes of bacteria (Gram-negative and Gram-positive species). They disrupt the function of the bacterial cell membrane or inhibit intracellular activity. The positive charge of AMPs was identified as a key factor for selective interaction with the bacterial versus human anionic membrane. Importantly, the hydrophobic part plays a key role in the effective interaction with the hydrophobic interior of the bacterial cell membrane [97]. After reaching a certain concentration, AMPs can induce pore formation or disrupt the function of bacterial organelle membranes after penetrating their interior [98]. Many AMPs inhibit protein and nucleic acid synthesis, inhibit enzyme activity, or induce apoptosis by generating reactive oxygen species. Examples of peptides with antimicrobial activity are shown in Table 4. 

Interestingly, AMPs also exhibit antiviral activity with several mechanisms of action. One of them is to combine negatively charged heparan sulfate on the viral surface and thus prevent the virus from docking [103], acting directly on the viral envelope by breaking it down or inhibiting viral gene expression [104]. Given the wide-ranging effects of AMPs, new applications are being sought. 

#### 2.4.2. AMPs as Cosmetic Ingredients

AMPs exert therapeutic effects and can support the pharmacotherapy of many diseases. Since they exhibit low toxicity to human cells and no induction of bacterial resistance to drugs, they have also found application as preservatives in the production of cosmetics [94].

Yun et al. investigated the structure of a peptide analogue, KR-12-pa, derived from the human antimicrobial peptide LL-37. It exhibited high antimicrobial activity, similar to that of LL-37, and low toxicity to human cells. The results of the study indicated that the MIC value (Table 2) against various species of bacteria and yeasts was much lower than that of commercial preservatives. In addition, KR-12-pa in cosmetic formulations showed stronger bactericidal activity than conventional preservatives. Importantly, when applied in cosmetics, it was found to have very low toxicity to human monocytes/macrophage-like cells [100].

Another example of peptides that can be used as natural cosmetic preservatives are animal peptides citropin 1.1 (GLFDVIKKVASVIGGL) and protegrin 1 (RGGRLCYCRRRFCVCVGR), whose antimicrobial activity was tested alone and in combination [101]. The results of the study indicated that, after 48 h of incubation, protegrin 1 exhibited similar or better antimicrobial properties against *Escherichia coli*, *Staphylococcus aureus*, *Pseudomonas aeruginosa*, *Candida albicans*, and *Aspergillus niger* than the commonly used agent benzalkonium chloride. Interestingly, a mixture consisting of citropin, protegrin, benzalkonium chloride, and citropin + protegrin 1:2 in 5 mL completely inhibited *C. albicans* and *A. niger* growth after only 6 h of incubation. Moreover, the growth of the other analyzed microorganisms was inhibited after 48 h of incubation.

Peptides of plant origin can also be used as natural cosmetic preservatives. Peptides with sequences GQLGEHGGAGMG (GG-12), GEHGGAGMGGGQFQPV (GV-16), EQGFLPGPEESGR (ER-13), RLARAGLAQ (RQ-9), YGNPVGGVGH (YH-10), and GNPVGGVGHGTTGT (GT-14) were identified in millet grains [92]. The antimicrobial activities of these peptides were tested against bacteria *Escherichia coli*, *Staphylococcus aureus*, *Listeria monocytogenes*, *Bacillus cereus*, *Salmonella enteritidis* and yeast *Candida albicans*. The peptides generally had antimicrobial properties, but no activity against *B. cereus* was observed (Table 1). Importantly, they had no cytotoxic effect on endothelial cells. This indicates that peptides can be used as natural cosmetic preservatives.

Given the increasing use of antibiotics, the growing resistance of microorganisms to antibiotics, and the greater consumer awareness of synthetic preservatives, new broad-spectrum preservatives are being sought. Non-toxic and harmless peptides may find application in the production of cosmetics and become substitutes for traditional preservatives in the future.

### 2.5. Other Compounds

Some cosmetic ingredients, e.g., moisturizing or greasing agents, also have antimicrobial activity. Such substances are represented by low molecular weight alcohols: ethanol or isopropanol [105]. Some bactericidal properties are also exhibited by lactic acid and polyols.

Lactic acid is commonly used to inhibit the growth of various pathogens. Its exact mechanism of action has not been elucidated. Some experimental results showed that 0.5% lactic acid completely inhibited the growth of *E. coli* and *L. monocytogenes* cells. It also caused protein leakage from *E. coli* and *Listeria* cells, and the amount of leakage after 6 h of exposure was 11.76 and 16.29 mg/mL, respectively [106].

Polyols used in low concentrations in cosmetics have a strong moisturizing effect on the skin; when used in higher concentrations, they can protect finished products against microbial contamination. Depending on the applied concentrations, polyols can replace preservatives or reduce their content in cosmetic products through synergistic action [20,107].

## 3. Conclusions

Antimicrobial compounds can limit microbial growth and development. The use of low concentrations of synthetic compounds may lead to the emergence of microbial resistance. Natural compounds can kill or slow down the growth of bacteria, algae, and fungi.

The production of natural antimicrobial compound-based cosmetics can have a positive impact on the development of the cosmetic industry given the increased consumer interest in natural products. Polyphenolic compounds, peptides, essential oils, or plant extracts can replace chemical compounds which currently are commonly used in cosmetic production. In addition to their antimicrobial properties, they can also act as antioxidant and anti-inflammatory compounds and impart a pleasant fragrance and texture to products. Products that contain natural compounds increasing their durability and consumer safety and have a positive impact on the environment are a promising element of the cosmetic industry.

## Figures and Tables

**Table 2:** Examples of phenolic compounds and their antimicrobial properties.

Phenolic Compound	Properties	The Concentration of the Compound Affecting Microorganisms	Reference
Catechuic acid	*Activity against C. acnes*	78 mM	[62]
Protocatechuic acid	*Growth inhibition in E. coli*, *S. aureus*, *S. typhimurium*, *E. coli*, *K. pneumoniae*, *and B. cereus*	0.3 mg/mL	[64]
p-Hydroxy benzoic, vanillic, caffeic, p-coumaric acids, oleuropein, quercetin acid	*Growth inhibition in Bacillus cereus*	0.4 mg/mL	[64]
Caffeic acid	*Growth inhibition in E. coli and K. pneumoniae; reduced aflatoxin production in A. flavus and A. parasiticus*	0.2 mg/mL	[64]
Vanillic acid	*Ability to destroy the cell wall of carbapenem-resistant E. hormaechei and prevention of biofilm formation by this bacterium; growth inhibition and reduced aflatoxin production in A. flavus and A. parasiticus*	0.2 mg/mL	[64,65]
Pyrogallol	*Reduction in required concentrations of antibiotics necessary to kill the entire C. albicans population; enhancement of antibiotic activity against S. aureus*	128 mg/mL	[66]
Gallic acid	*Enhancement of antibiotic activity against S. aureus*	128 mg/mL	[66]
Quercetin	*Growth inhibition and destruction of S. aureus*, *P. aeruginosa*, *P. vulgaris*, *E. coli*, *S. flexneri*, *and L. casei var. Shirota; inhibitory effect against drug-resistant E. coli or carbapenem-resistant P. aeruginosa*	20 µg/mL	[67]
Kaempferol	*Inhibition of S. aureus biofilm formation*	64 mg/mL	[68]
Apigenin and luteolin	*Growth inhibition in E. coli and P. aeruginosa*	500–1000 µg/mL	[69]
3′-demethoxy-6-O-demethylisoguaiacin lignan	*Growth inhibition in methicillin-resistant S. aureus*, *E. faecalis*, *E. coli*, *and E. cloacae*	25 µg/mL 12.5 µg/mL 50 µg/mL 12.5 µg/mL	[70]
Dihydroguaiaretic acid and 4-epi-larreatricin	*Growth inhibition in S. aureus MR and E. cloacae*	50 µg/mL	[70]

**Table 3:** Essential oils with antimicrobial properties.

Essential Oil	Strains	MIC ^1^ (μg/mL)	Reference
*Ferulago stellata* Boiss	*Escherichia coli* *Enterococcus faecalis* *Salmonella typhi* *Pseudomonas aeruginosa* *Staphylococcus epidermidis* *Staphylococcus aureus* *Listeria monocytogenes* *Candida albicans*	0.78 3.12 1.56 0.78 1.56 1.56 0.78 0.78	[81]
*Elsholtzia beddomei* C. B. Clarke ex Hook	*Escherichia coli* *Pseudomonas aeruginosa*, *Enterobacter aerogenes* *Staphylococcus aureus* *Staphylococcus epidermidis* *Bacillus subtilis* *Candida albicans*	7.81 7.81 7.81 3.91 3.91 3.91 15.62	[82]
*Rosa* × *damascena* from Sedeh	*Staphylococcus epidermidis* *Staphylococcus aureus* *Streptococcus pyogenes* *Bacillus subtilis* *Klebsiella pneumoniae* *Shigella dysenteriae * *Pseudomonas aeruginosa * *Salmonella paratyphi-A serotype* *Escherichia coli* *Aspergillus brasiliensis* *Candida albicans*	250 500 <15.63 250 250 250 62.50 125 250 1000 250	[83]
*Rosa* × *damascena* from Javinan	*Staphylococcus epidermidis* *Staphylococcus aureus* *Streptococcus pyogenes* *Bacillus subtilis* *Klebsiella pneumoniae* *Shigella dysenteriae* *Pseudomonas aeruginosa* *Salmonella paratyphi-A serotype* *Escherichia coli* *Aspergillus brasiliensis* *Candida albicans*	250 500 <15.63 250 250 250 125 250 250 1000 62.50	[83]
Lettuce leaf basil (*Ocimum basilicum* L.)	*Escherichia coli* *Staphylococcus aureus*	0.625 0.625	[84]
Lettuce leaf basil (*Ocimum basilicum* L.) elicited with 100 µM jasmonic acid	*Escherichia coli* *Staphylococcus aureus*	0.312 0.156	[84]
Citron essential oil	*Bacillus subtilis* *Staphylococcus aureus* *Micrococcus luteus * *Escherichia coli*	0.625 * 0.625 * 1.25 * 2.5 *	[85]
*Satureja montana*	*Staphylococcus aureus* *Streptococcus pyogenes* *Streptococcus mutans* *Streptococcus salivarius* *Streptococcus sanguinis* *Pseudomonas aeruginosa* *Enterococcus faecalis*	26.62 116.67 60.00 56.67 23.33 23.33 53.33	[86]
*Mentha pulegium* L.	*Staphylococcus aureus* *Streptococcus pyogenes* *Streptococcus mutans* *Streptococcus salivarius* *Streptococcus sanguinis* *Pseudomonas aeruginosa* *Enterococcus faecalis*	626.67 620.00 630.00 593.33 620.00 850.00 1191.67	[86]

^1^ Minimum inhibitory concentration; * mg/mL.

**Table 4:** Peptides with antimicrobial properties.

Peptide	Strains	MIC ^1^ (μg/mL)	Reference
LPcin-YK3	*Staphylococcus aureus* *Escherichia coli* *Porphyromonas gingvalis* *Streptococcus mutans* *Propionibacterium agnes* *Candida albicans*	0.62 1.25 0.62 1.25 1.25 10.0	[99]
KRIVQRIKDFLR-pa (KR-12-pa)	*Escherichia coli* *Salmonella typhimurium* *Pseudomonas aeruginosa* *Staphylococcus ureus* *Bacillus subtilis* *Staphylococcus epidermidis* *Candida glabrata*	1.6 1.6 3.2 3.2 3.2 3.2 12.8	[100]
RGGRLCYCRRRFCVCVGR (protegrin 1)	*Escherichia coli* *Staphylococcus ureus* *Pseudomonas aeruginosa* *Candida albicans* *Aspergillus niger*	4 8 8 8 64	[101]
GLFDVIKKVASVIGGL citropin 1.1	*Escherichia coli* *Staphylococcus ureus* *Pseudomonas aeruginosa* *Candida albicans* *Aspergillus niger*	128 8 256 16 32	[101]
GQLGEHGGAGMG (GG-12)	*Staphylococcus aureus* *Listeria monocytogenes*	250 250	[92]
GEHGGAGMGGGQFQPV (GV-16)	*Listeria monocytogenes*	62.5	[92]
EQGFLPGPEESGR (ER-13)	*Listeria* *monocytogenes*	125	[92]
RLARAGLAQ (RQ-9)	*Escherichia coli* *Salmonella enteritidis* *Listeria monocytogenes* *Candida albicans*	250 15.62 15.62 15.62	[92]
YGNPVGGVGH (YH-10)	*Listeria monocytogenes*	125	[92]
GNPVGGVGHGTTGT (GT-14)	*Escherichia coli* *Staphylococcus aureus* *Listeria monocytogenes*	250 250 250	[92]
FFPRVLPLANKFLPTIYCALPKSVGN	*Staphylococcus aureus* *Pseudomonas aeruginosa* *Escherichia coli*	>88 >2560 6–44	[102]
GLWETIKTTGKSIALNLLDKIKCKIAGGCPP	*Staphylococcus aureus* *Pseudomonas aeruginosa* *Escherichia coli*	>78 >78 5–10	[102]

^1^ Minimum inhibitory concentration.

## Data Availability

Not applicable.
